# Supplementary material for: Improved figure of merit (z) at low temperatures for superior thermoelectric cooling in Mg3(Bi,Sb)2
Source: Nat Commun. 2023 Aug 15;14:4932. doi: 10.1038/s41467-023-40648-5 (PMC10427716; doi:10.1038/s41467-023-40648-5)
Supplement: Supplementary file 1 — Supplementary Information [file 41467_2023_40648_MOESM1_ESM.pdf]

Supplementary Materials

Improved Figure of Merit (z) at Low Temperatures for Superior Thermoelectric Cooling in $Mg_3(Bi,Sb)_2$

Nan Chen[1,2], Hangtian Zhu[1*], Guodong Li[1], Zhen Fan[1], Xiaofan Zhang[1,2], Jiawei Yang[1,2], Tianbo Lu[1], Qiulin Liu[1,2], Xiaowei Wu[1,2], Yuan Yao[1], Youguo Shi[1], Huaizhou Zhao[1*]

[1]*Beijing National Laboratory for Condensed Matter Physics, Institute of Physics, Chinese Academy of Sciences, Beijing 100190, China.*
[2]*College of Materials Science and Opto-Electronic Technology, University of Chinese Academy of Sciences, Beijing 100049, China.*

* Corresponding authors, email address: htzhu@iphy.ac.cn; hzhao@iphy.ac.cn

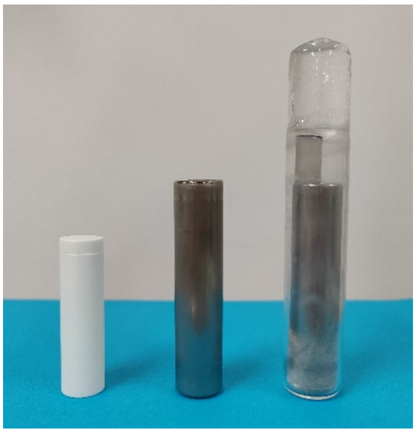

Supplementary Fig. 1. The pictures of ZrO_2 crucible, sealed Ta tube containing ZrO_2 crucible, and sealed quartz crucible containing sealed Ta tube.

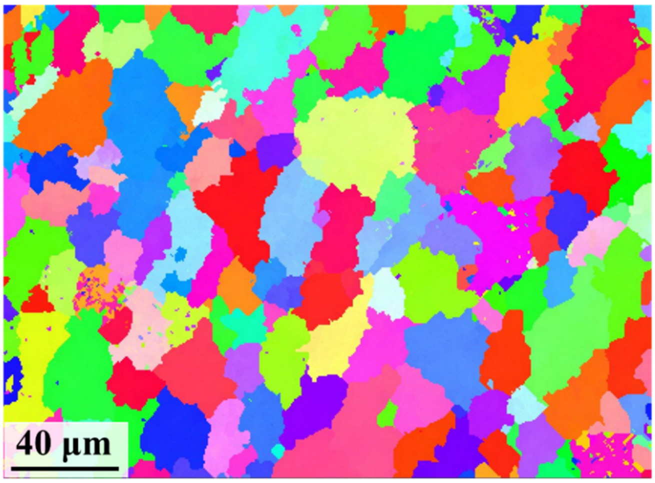

Supplementary Fig. 2. The EBSD image of $Mg_{3.2}Bi_{1.4975}Sb_{0.5}Te_{0.0025}$ prepared by SPS.

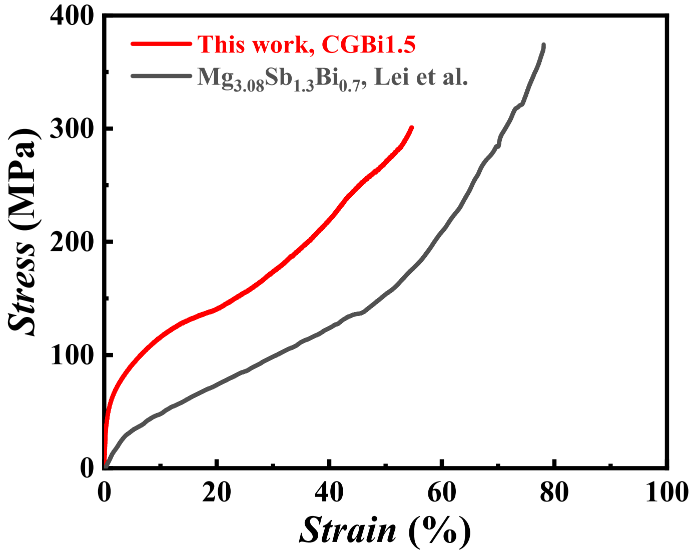

Supplementary Fig. 3. The compressive stress-strain curves of CGBi1.5 bulk materials, comparable to literature data[1].

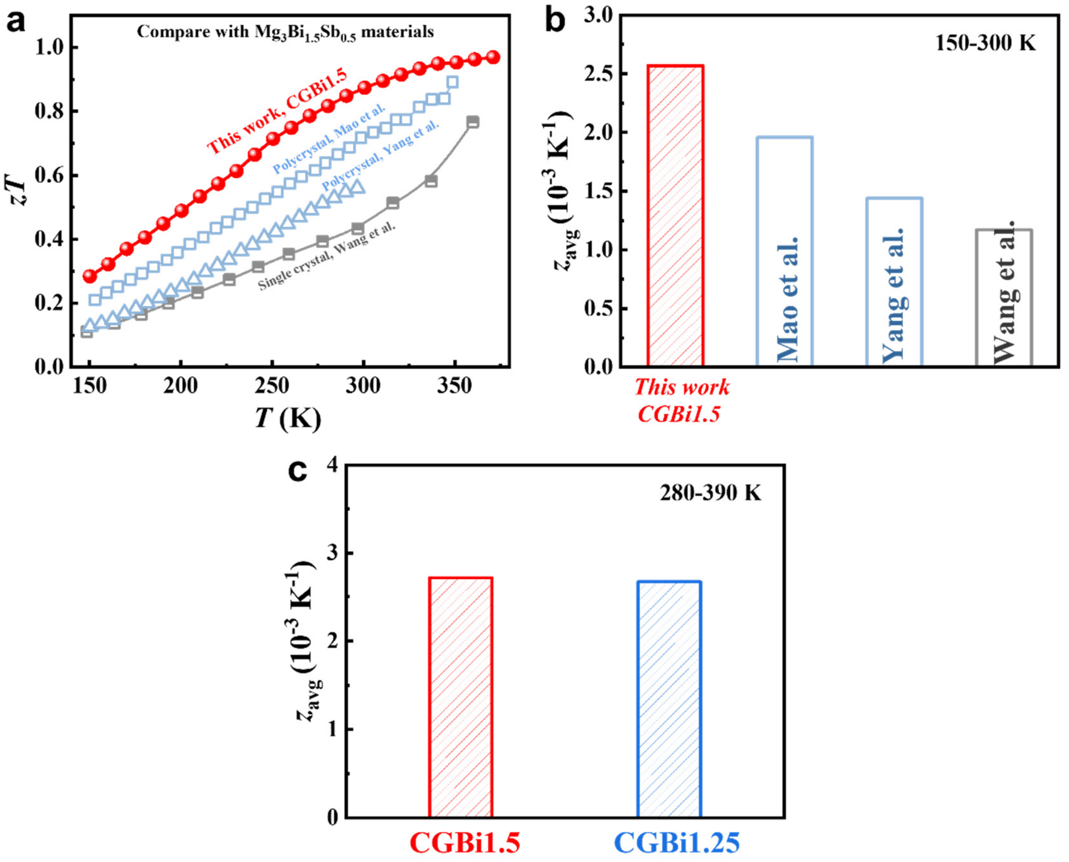

Supplementary Fig. 4. Thermoelectric properties of CGBi1.5 and CGBi1.25 materials. (a) The measured zT of as-grown CGBi1.5 sample as a function of temperature, compared to literatures[2-4]. (b) The comparison of average z (z_{avg}) between $Mg_3Bi_{1.5}Sb_{0.5}$ based materials at temperature range of 150 - 300 K[2-4]. (c) z_{avg} of CGBi1.5 and CGBi1.25 materials in this work.

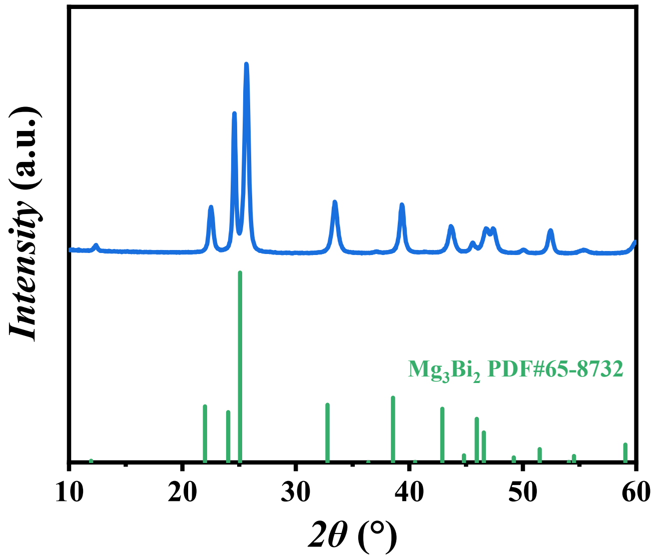

Supplementary Fig. 5. The XRD pattern of CGBi1.25 crystals.

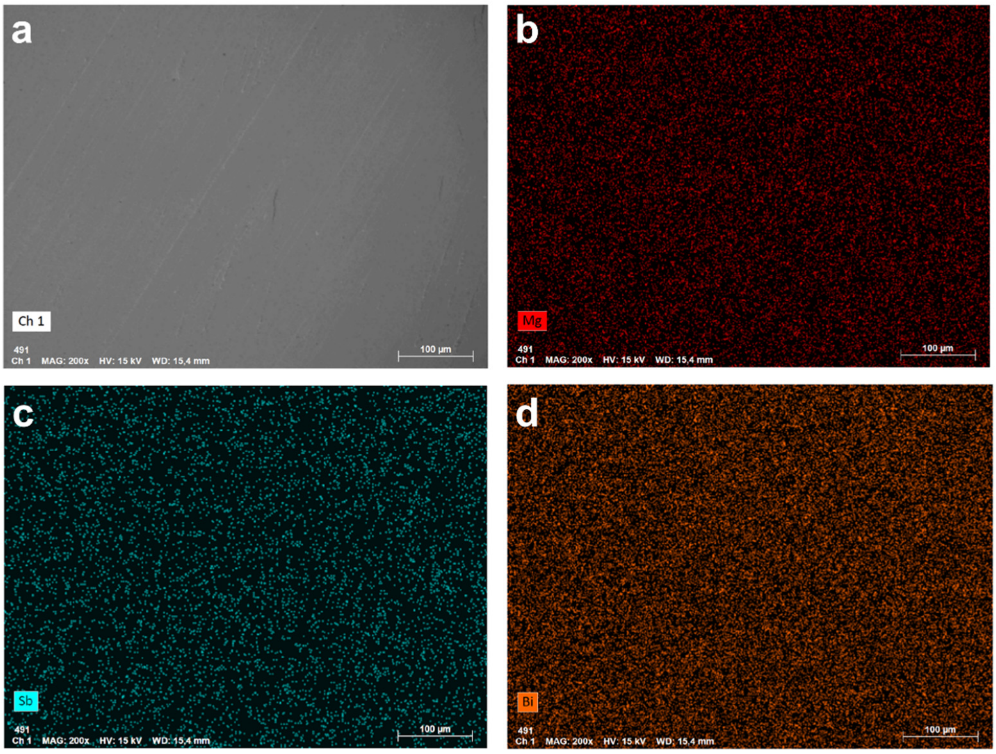

Supplementary Fig. 6. Scanning electron microscopy (SEM) and energy dispersive spectrometer (EDS) analysis for CGBi1.5 samples. (a) SEM image and elemental mappings for (b) Mg, (c) Sb and (d) Bi.

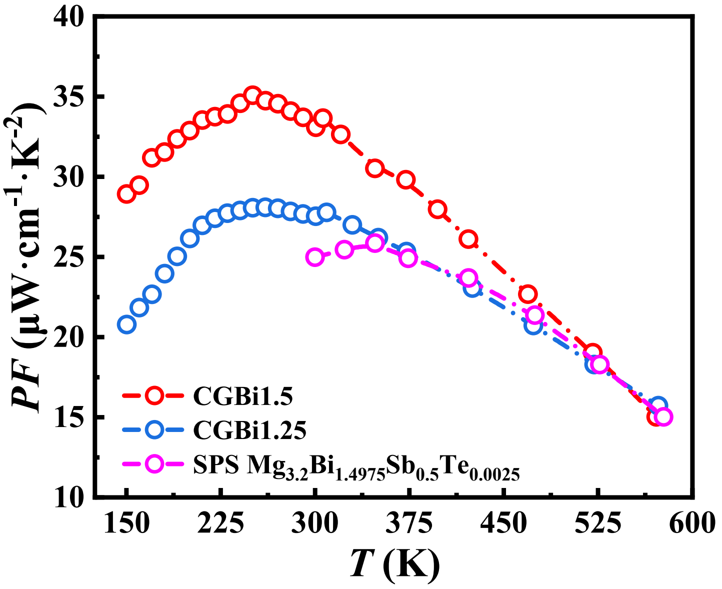

Supplementary Fig. 7. The power factor of as-grown CGBi1.5, CGBi1.25, and SPS $Mg_{3.2}Bi_{1.4975}Sb_{0.5}Te_{0.0025}$ samples.

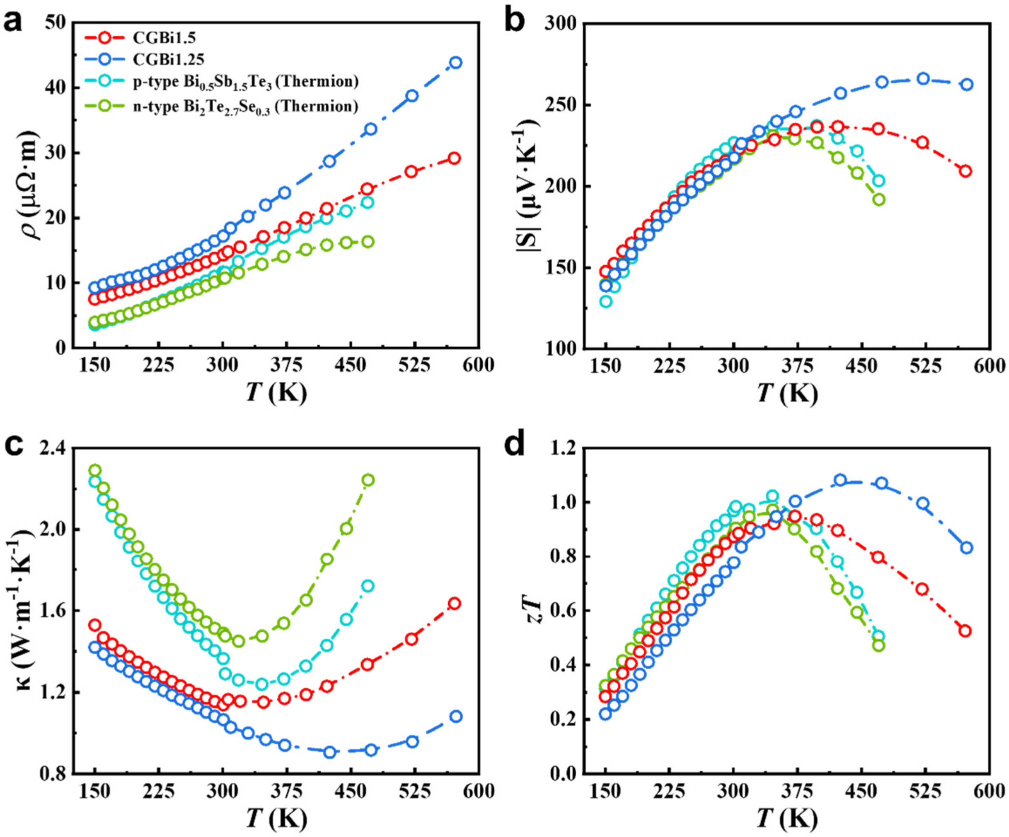

Supplementary Fig. 8. Thermoelectric properties of CGBi1.5, CGBi1.25, and commercial Bi_2Te_3 materials from Thermion Company. (a) Electrical resistivity, (b) Seebeck coefficient, (c) thermal conductivity, and (d) *zT*.

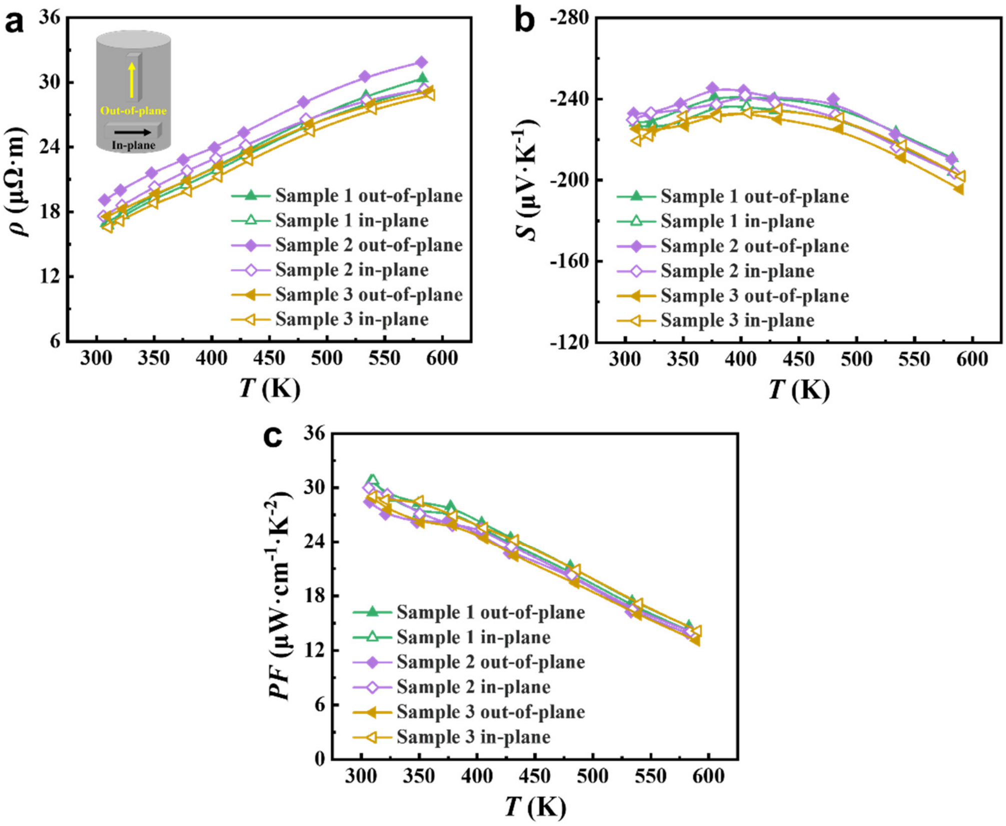

Supplementary Fig. 9. The electrical transport properties as a function of temperature along the out-of-plane and in-plane directions in CGBi1.5 bulk samples from three different batches. (a) Electrical resistivity, (b) Seebeck coefficient and (c) power factor. The almost identical experimental data in two directions not only shows the isotropic properties of the coarse grain materials, but also indicates the uniformity of the samples.

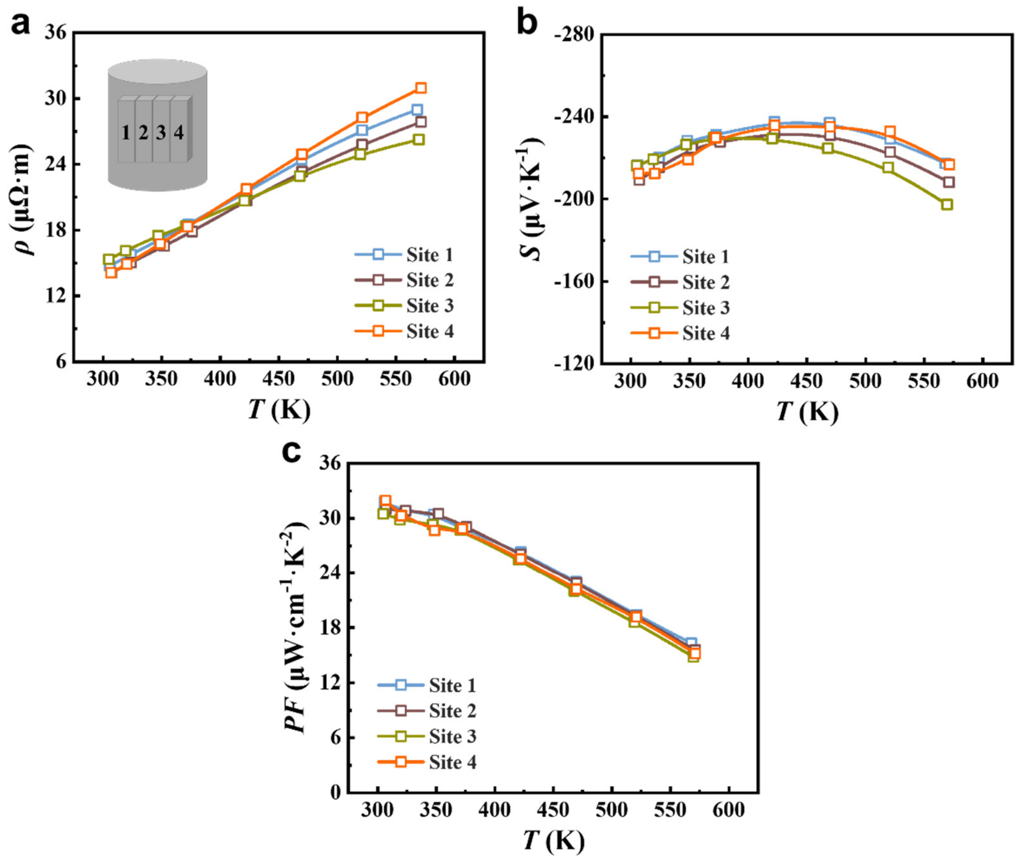

Supplementary Fig. 10. Comparison of the electrical transport properties among different parts of the as-grown CGBi1.5 bulk ingot. (a) Electrical resistivity, (b) Seebeck coefficient and (c) power factor. Four bars with dimensions of ~ 2.5×2.5×8 mm^3 were cut in the out-of-plane direction from one CGBi1.5 bulk materials. No significant difference in the electrical transport properties of the four bars was observed.

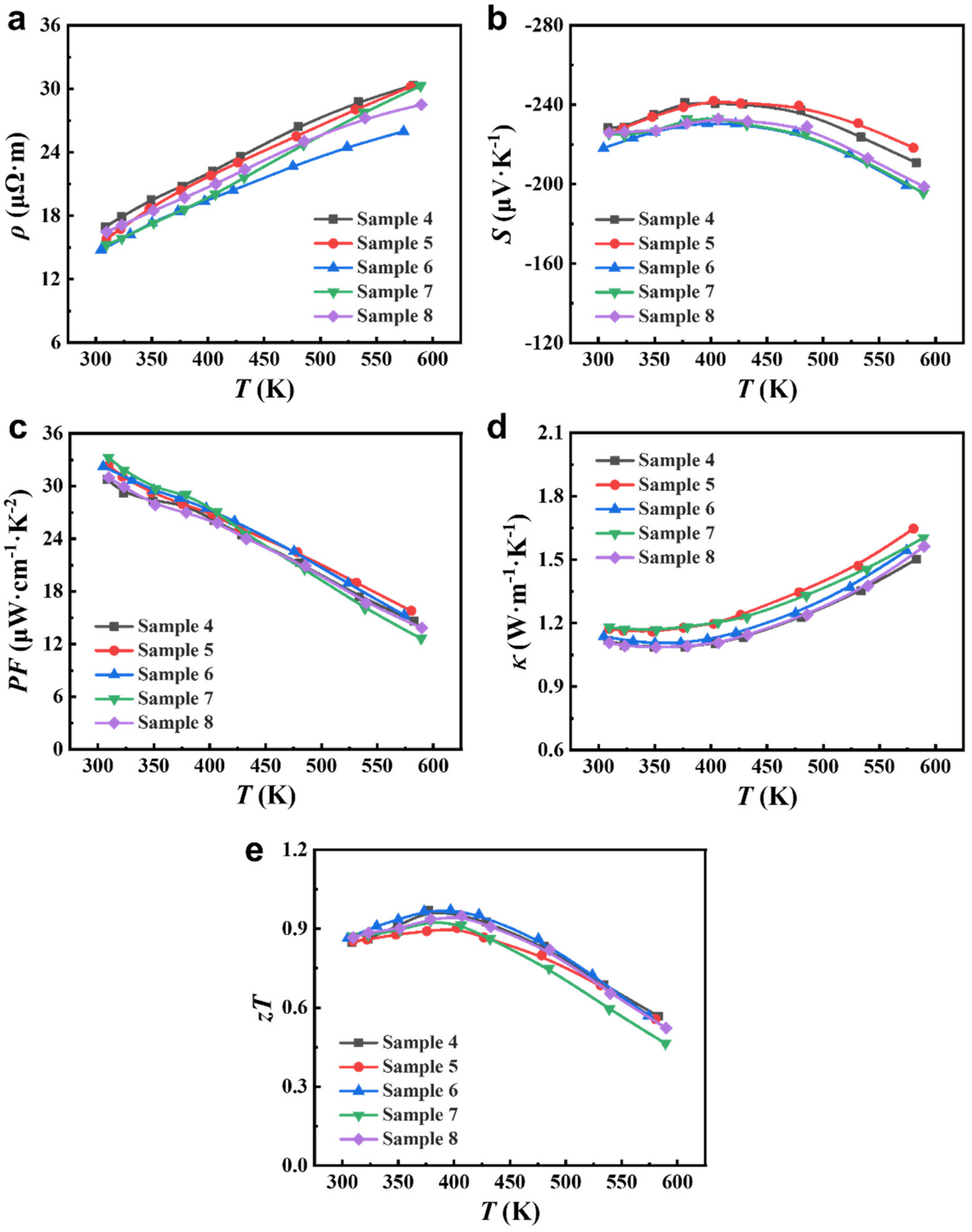

Supplementary Fig. 11. Thermoelectric properties of samples of CGBi1.5 from different batches in this work demonstrate the good reproducibility. (a) Electrical resistivity, (b) Seebeck coefficient, (c) power factor, (d) thermal conductivity and (e) *zT*.

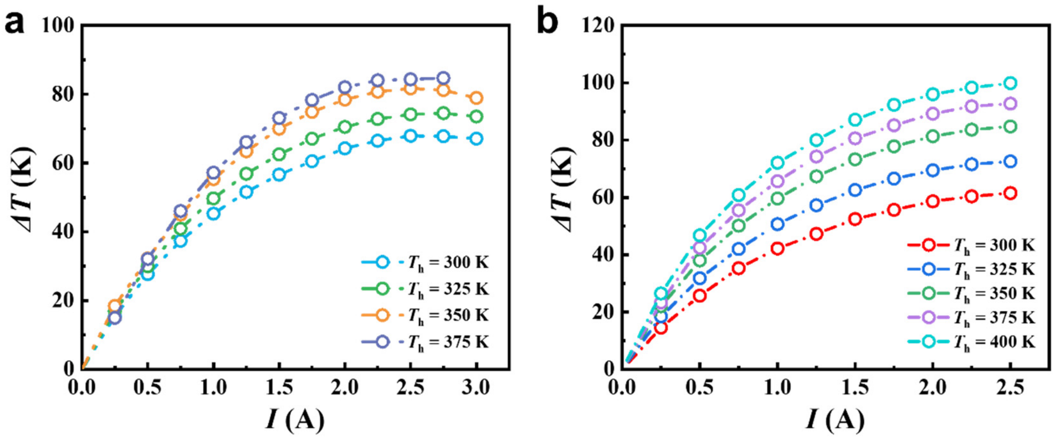

Supplementary Fig. 12. Temperature differences of (a) CGBi1.5/$Bi_{0.5}Sb_{1.5}Te_3$ and (b) CGBi1.25/$Bi_{0.5}Sb_{1.5}Te_3$ modules as a function of current.

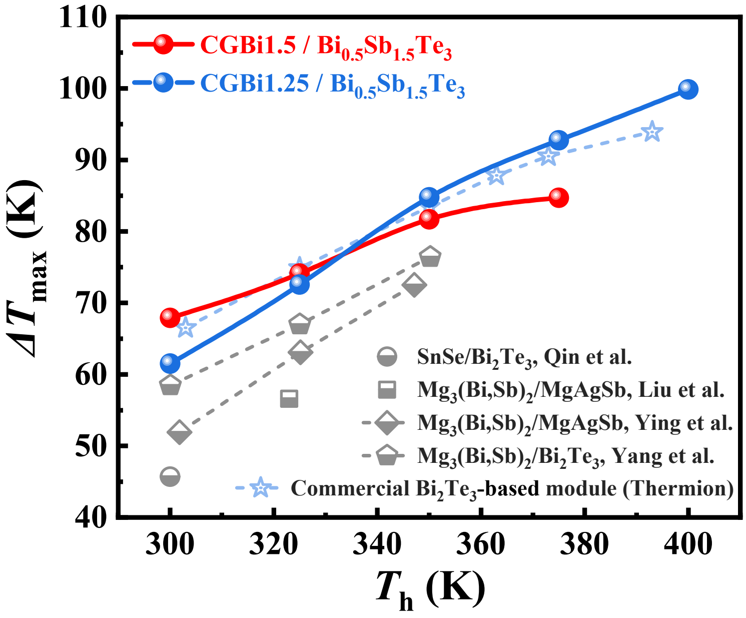

Supplementary Fig. 13. Comparison of the maximum ΔT of real cooling devices, including our $Mg_3(Bi,Sb)_2$/$Bi_{0.5}Sb_{1.5}Te_3$ modules and the modules fabricated by n-$Mg_3(Bi,Sb)_2$ systems[2,5,6], SnSe/Bi_2Te_3[7], and commercial Bi_2Te_3 materials.

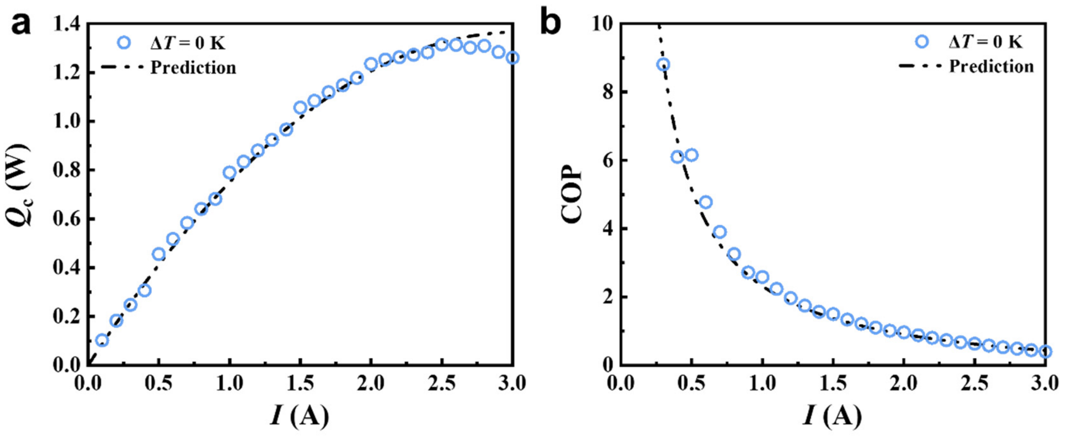

Supplementary Fig. 14. Measured (a) Q_c and (b) COP of commercial Bi_2Te_3-based module as a function of current.

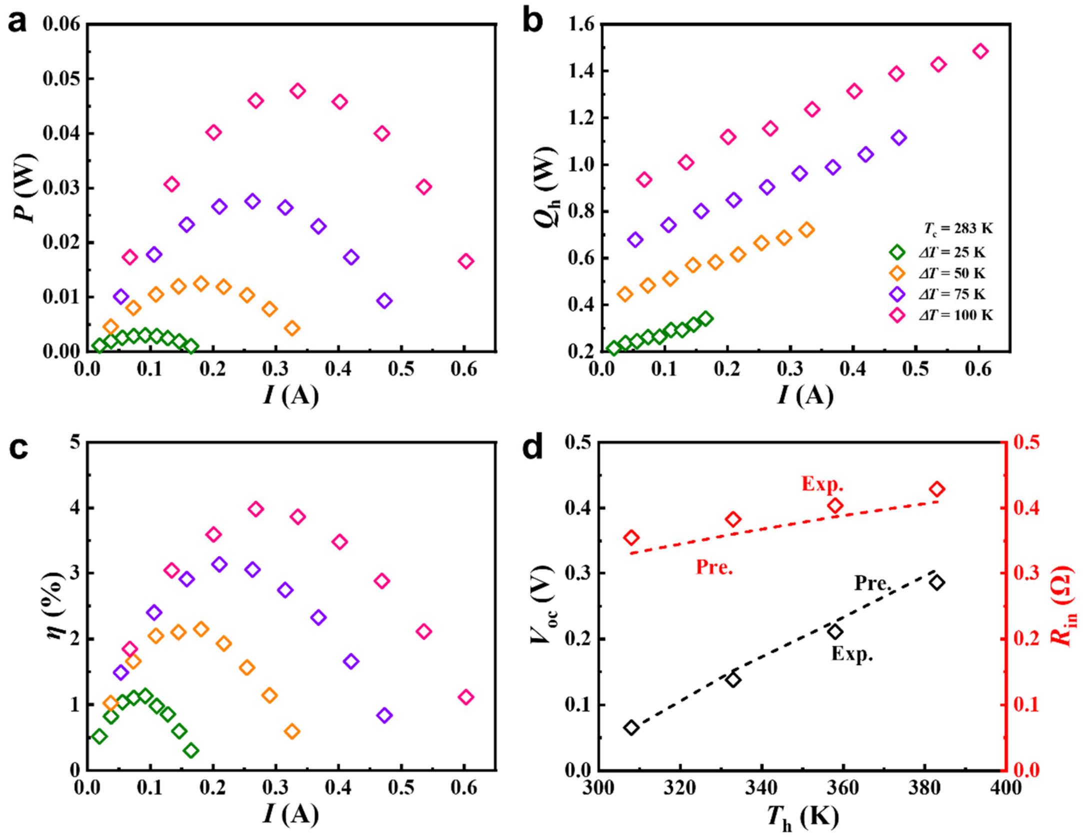

Supplementary Fig. 15. Power generation properties of CGBi1.5/$Bi_{0.5}Sb_{1.5}Te_3$ module. (a) Output power (P), (b) heat-flow from hot-side (Q_h), (c) conversion efficiency (η), and (d) open-circuit voltage (V_{oc}) and internal resistance (R_{in}).

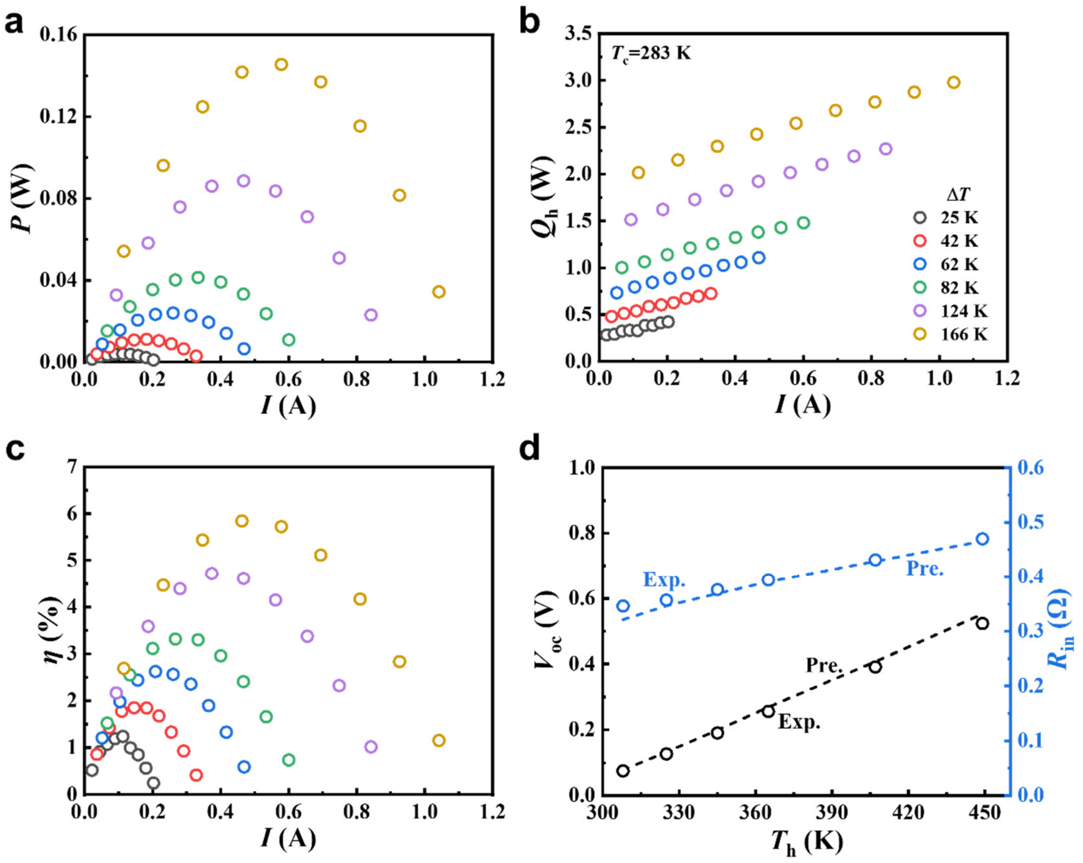

Supplementary Fig. 16. Power generation properties of CGBi1.25/$Bi_{0.5}Sb_{1.5}Te_3$ module. (a) Output power (P), (b) heat-flow from hot-side (Q_h), (c) conversion efficiency (η), and (d) open-circuit voltage (V_{oc}) and internal resistance (R_{in}).

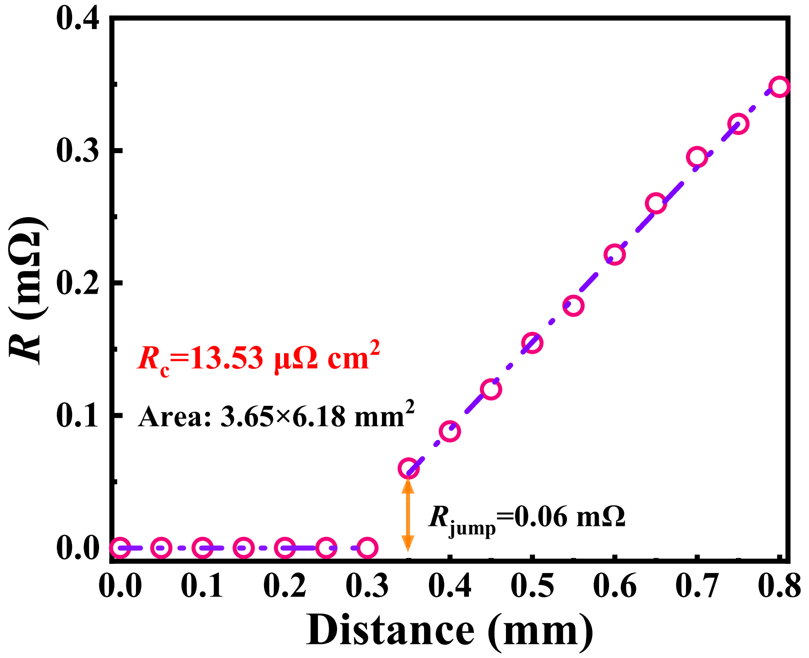

Supplementary Fig. 17. Contact resistivity of Mg_2Cu/CGBi1.5 interface.

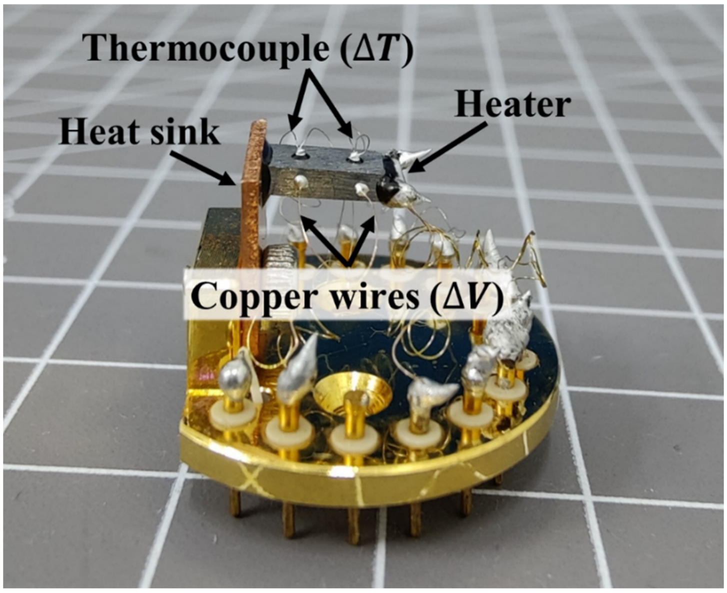

Supplementary Fig. 18. Measurement setup for low-temperature (150 - 370 K) Seebeck coefficient and thermal conductivity measurement by steady state method.

Supplementary Table 1. Densities of as-grown CGBi1.5 and CGBi1.25 samples, and the calculated densities from the two end members assuming a linear relation[8].

Samples	CGBi1.5		CGBi1.25	
	Measured	Calculated	Measured	Calculated
Density (g·cm^{-3})	5.36	5.39	5.14	5.16

Supplementary Table 2. Crystallographic data for the CGBi1.5 material from the Rietveld refinement against XRD data (*nb.* the lightly doped Te element was not included in the refinement).

**Chemical Formula**	$\mathbf{Mg_{2.922(13)}Bi_{1.5}Sb_{0.5}}$
Crystal System	Trigonal
Space Group	*P*-3m1
Lattice parameters / Å	4.6652(1)
(α=β=90°,	4.6652(1)
γ=120°)	7.4058(2)
Cell volume / Å^3	139.585(8)
Formula weight / g mol^{-1}	445.376
Formula units, Z	1
Calculated density / g cm^{-3}	5.298
No. of observations	11370
No. of parameters	25
χ^2	5.883
Rp	0.0729
wRp	0.0954

Supplementary Table 3. Atomic parameters of CGBi1.5 from the Rietveld refinement against XRD data (see also Table S2).

**Atom**	**Wyckoff symbol**	***x***	***y***	***z***	**100 × U_{iso}/Å^2 (Equivalent)**	**Thermal Displacement Parameters, U_{ij}/Å^2**	**Occupancy**
Mg1	1*a*	0	0	0	2.35	U^{11}=U^{22}=0<br>U^{33}=0.076(9)<br>U^{12}=U^{13}=U^{23}=0	0.922(13)
Mg2	2*d*	0.3333	0.6667	0.6311(5)	1.71	U^{11}=U^{22}=0.0136(23)<br>U^{33}=0.024(3)<br>U^{12}=0.0068(11) U^{13}=U^{23}=0	1.0
Bi	2*d*	0.3333	0.6667	0.2258(1)	1.33(3)	-	0.75
Sb	2*d*	0.3333	0.6667	0.2258(1)	1.33(3)	-	0.25

Supplementary Table 4. The fitting parameters used in the $\mu_H \sim d$ curve. m^* of 0.36 m_e was used for fitting.

**Grain sizes (*d*, μm)**	**Carrier mobility (μ_H, $cm^2 \cdot V^{-1} \cdot s^{-1}$)**	**Reference**
5	78	
10	133	[6]
20	172	
50	169	[9]
9000	220	[4]
25	165	This work, SPS sample
800	210	This work, CGBi1.5

References:

1. Lei J, *et al.* Efficient lanthanide Gd doping promoting the thermoelectric performance of Mg_3Sb_2-based materials. *J. Mater. Chem. A* **9**, 25944-25953 (2021).
2. Yang J, *et al.* Next-generation thermoelectric cooling modules based on high-performance $Mg_3(Bi,Sb)_2$ material. *Joule* **6**, 193-204 (2022).
3. Mao J, *et al.* High thermoelectric cooling performance of n-type Mg_3Bi_2-based materials. *Science* **365**, 495-498 (2019).
4. Wang QQ, *et al.* In-Situ Loading Bridgman Growth of $Mg_3Bi_{1.49}Sb_{0.5}Te_{0.01}$ Bulk Crystals for Thermoelectric Applications. *Adv. Electron. Mater.* **8**, 2101125 (2022).
5. Liu Z, Gao W, Oshima H, Nagase K, Lee CH, Mori T. Maximizing the performance of n-type Mg_3Bi_2 based materials for room-temperature power generation and thermoelectric cooling. *Nat Commun* **13**, 1120 (2022).
6. Ying P, *et al.* A robust thermoelectric module based on MgAgSb/$Mg_3(Sb,Bi)_2$ with a conversion efficiency of 8.5% and a maximum cooling of 72 K. *Energy Environ. Sci.* **15**, 2557-2566 (2022).
7. Qin B, *et al.* Power generation and thermoelectric cooling enabled by momentum and energy multiband alignments. *Science* **373**, 556-561 (2021).
8. Wood M, Kuo JJ, Imasato K, Snyder GJ. Improvement of Low-Temperature *zT* in a Mg_3Sb_2 - Mg_3Bi_2 Solid Solution via Mg-Vapor Annealing. *Adv Mater* **31**, e1902337 (2019).
9. Imasato K, Kang SD, Snyder GJ. Exceptional thermoelectric performance in $Mg_3Sb_{0.6}Bi_{1.4}$ for low-grade waste heat recovery. *Energy Environ. Sci.* **12**, 965-971 (2019).
